# Pim-selective inhibitor DHPCC-9 reveals Pim kinases as potent stimulators of cancer cell migration and invasion

**DOI:** 10.1186/1476-4598-9-279

**Published:** 2010-10-19

**Authors:** Niina M Santio, Riitta L Vahakoski, Eeva-Marja Rainio, Jouko A Sandholm, Sanna S Virtanen, Michelle Prudhomme, Fabrice Anizon, Pascale Moreau, Päivi J Koskinen

**Affiliations:** 1 Turku Centre for Biotechnology, University of Turku and Åbo Akademi University, Turku, Finland; 2 Department of Biology, University of Turku, Finland; 3 Institute of Biomedicine and Anatomy, University of Turku, Finland; 4 Drug Discovery Graduate School, Finland; 5 Clermont Université, Université Blaise Pascal, SEESIB, Clermont-Ferrand, France; 6 CNRS, UMR6504, Aubière, France

## Abstract

**Background:**

Pim family kinases are small constitutively active serine/threonine-specific kinases, elevated levels of which have been detected in human hematopoietic malignancies as well as in solid tumours. While we and others have previously shown that the oncogenic Pim kinases stimulate survival of hematopoietic cells, we now examined their putative role in regulating motility of adherent cancer cells. For this purpose, we inhibited Pim kinase activity using a small molecule compound, 1,10-dihydropyrrolo[2,3-*a*]carbazole-3-carbaldehyde (DHPCC-9), which we had recently identified as a potent and selective inhibitor for all Pim family members.

**Results:**

We now demonstrate that the Pim kinase inhibitor DHPCC-9 is very effective also in cell-based assays. DHPCC-9 impairs the anti-apoptotic effects of Pim-1 in cytokine-deprived myeloid cells and inhibits intracellular phosphorylation of Pim substrates such as Bad. Moreover, DHPCC-9 slows down migration and invasion of cancer cells derived from either prostate cancer or squamocellular carcinoma patients. Silencing of Pim expression reduces cell motility, while Pim overexpression enhances it, strongly suggesting that the observed effects of DHPCC-9 are dependent on Pim kinase activity. Interestingly, DHPCC-9 also abrogates NFATc-dependent migration of cancer cells, implying that NFATc factors mediate at least part of the pro-migratory effects of Pim kinases.

**Conclusions:**

Altogether, our data indicate that DHPCC-9 is not only a powerful tool to investigate physiological effects of the oncogenic Pim family kinases, but also an attractive molecule for drug development to inhibit invasiveness of Pim-overexpressing cancer cells.

## Background

The mammalian Pim family of serine/threonine-specific kinases consists of three highly homologous proteins, Pim-1, Pim-2 and Pim-3, whose functions and expression patterns are partially overlapping [1,2]. Crystallization studies have revealed that Pim kinases constitutively reside in an active conformation [3], so that their activities are mainly regulated at the level of expression. In hematopoietic cells *pim *expression is transiently induced by a number of cytokines including several types of interleukins (IL; [4-6]). When overexpressed in mice, Pim kinases are oncogenic and can enhance lymphomagenesis, especially in collaboration with other oncoproteins such as Myc family members, Bcl-2 or Gfi-1 [7-10]. Upregulated expression levels for Pim kinases have been observed not only in human leukemias and lymphomas [11], but also in several types of solid tumors such as prostate, colon, oral, hepatic and pancreatic cancers (reviewed in [12,13]).

There are many ways how Pim kinases contribute to tumorigenesis by promoting proliferation and preventing apoptosis (reviewed in [13,14]). In hematopoietic cells, we have previously shown that Pim-1 stimulates activities of several transcription factors including c-Myb [15], NFATc1 [16] and the RUNX family proteins [17], and thereby enhances production of cytokines and other survival factors. In addition, all Pim family members inhibit apoptosis by phosphorylating and thereby inactivating the pro-apoptotic Bad protein [18-20]. However, the physiological role of Pim kinases in development of solid tumors has remained fairly elusive.

The emerging importance of Pim kinases in human tumorigenesis has raised growing interest to develop small molecule inhibitors for them. Several different classes of Pim inhibitors have recently been reported (reviewed in [13]), but only a few of them have been tested in cell-based assays or animal models to demonstrate anticancer activity [21-27]. In addition, only a few of them are effective against all Pim family kinases [21,25-27]. Due to functional redundancy [2], simultaneous targeting of all Pim kinases can be of advantage in treating cancer patients. Yet no severe side effects are expected, since mice lacking all three Pim family members are just slightly deficient in their growth responses, but otherwise viable and fertile with a normal life span [2].

In this study, we have further analysed the cellular effects of 1,10-dihydropyrrolo[2,3-*a*]carbazole-3-carbaldehyde (DHPCC-9) that we recently identified as a potent and selective inhibitor against all three Pim family kinases [21]. We now demonstrate that this inhibitor can efficiently block several cellular functions of Pim kinases. Furthermore, using this inhibitor along with RNA interference or protein overexpression, we have been able to reveal an as yet unrecognized role for all Pim family kinases in promoting migration and invasion of adherent cancer cells.

## Methods

### Cell lines and culture conditions

The murine IL-3-dependent myeloid FDCP1 cell lines and the human head and neck squamous cell carcinoma cell line UT-SCC-12 have been previously described [28,29]. FDCP1 cell lines and the human androgen-independent prostate epithelial adenocarcinoma cell line PC-3 (American Type Culture Collection) were maintained in RPMI-1640 medium, while UT-SCC-12 cells were cultured in DMEM medium with 1% non essential amino acids. All media were supplemented with 10% fetal bovine serum. 10% WEHI-conditioned medium was used as the source of IL-3 for FDCP1 cell lines.

### Cell viability assays

For MTT assays, cell cultures were incubated for 4 h with 0,5 mg/ml 3-(4,5-dimethylthiazol-2-yl)-2,5-diphenyl tetrazolium bromide (MTT) reagent (Sigma-Aldrich). Formazan crystals formed during the assays were dissolved by acidic isopropanol. Optical densities were determined by spectrophotometry (Multiscan MCC/340, Labsystems). Alternatively, cells were stained with Trypan blue (Sigma-Aldrich) and live cells excluding the dye were counted.

### Western blotting

Cell pellets were resuspended and lysed in NP-40 lysis buffer (50 mM Tris, pH 7.5, 10% glycerol, 100 mM NaCl, 1 mM EDTA, 1% Nonidet P-40 plus protease and phosphatase inhibitors). After clearing the lysates, protein concentrations were measured with D_C _Protein Assay (Bio-Rad). Twenty to hundred microgram aliquots of protein were separated by SDS-PAGE, immobilized onto PVDF-membrane (Millipore) and incubated with anti-Pim-1 (12H8; Santa Cruz), anti-Pim-2 (D1D2; Cell Signaling Technology for human protein or HPA000285; Atlas Antibodies for murine protein), anti-Pim-3 (D17C9; Cell Signaling Technology), anti-V5 (Invitrogen), anti-Flag or anti-GAPDH (Sigma-Aldrich) antibodies. Chemiluminescence reactions were generated by either Amersham™ ECL Plus (GE Healthcare) or Pierce^® ^ECL (Thermo Scientific) reagents. The signal intensities were quantified by MCID M5+ Image Analyzer (InterFocus, UK).

### Cell-based phosphorylation assays

FDCP1 derivatives were transiently transfected with 10 μg of the GST-Bad expression vector (pEBG-mBad; [19]) using the GenePulser II electroporator (Bio-Rad). Cells were lysed in buffer containing 10 mM Tris, pH 7.5, 150 mM NaCl, 0,5 mM EDTA, 1% Triton X-100, 10% glycerol and phosphatase inhibitors. Cleared lysates containing 60 μg aliquots of protein were used to purify GST-Bad protein with glutathione Sepharose beads (GE Healthcare) at 4°C. The precipitates were fractionated by SDS-PAGE and subjected to Western blotting with anti-Bad and anti-phospho-Bad (Ser^112^) antibodies (Cell Signaling Technology).

### Lipofections

For RNA interference, PC-3 cells were transfected with short interfering RNAs (siRNAs) and Oligofectamine™ (Invitrogen). Non-targeting control siRNA (D-001810-01-20) or specific siRNAs targeting either *pim-1 *or *pim-2 *(Dharmacon) were used at 100 nM concentration. These ON-TARGETplus siRNAs have been specifically designed to reduce off-target effects that may lead to toxicity or false phenotypes.

For overexpression, Fugene transfection reagents (Roche) were used to transfect PC-3 cells with 1 μg of the pcDNA3.1/V5-HisC vector or its derivatives expressing human *pim *genes (N.M. Santio, M. Varjosalo, J. Taipale and P.J. Koskinen, manuscript in preparation), or 0,25 μg of the pBJ5 vector or pBJ5-NFATc1-FLAG obtained from S.N. Ho (Stanford University, CA).

### Wound Healing assays

Wound healing assays were performed on 24-well plates either manually or automatically. In manually performed assays, different concentrations of DHPCC-9 were used, while DMSO concentration was maintained at 0,1%. Scratch wounds were made with a sterile 10 or 200 μl pipette tip. Photographs were taken using the Zeiss Stereo Lumar-V12 microscope with the AxioVision Rel.4.8 software. Percentages of wound healing were calculated by the ImageJ software (Wayne Rasband, NIH, USA) and the approximate edges of the wounds were manually marked to figures with straight lines.

For automatic assays with the WoundMaker™ and the IncuCyte™ systems (Essen Instruments), the scratch wounds were made with 10 μl sterile pipette tips, after which fresh culture medium containing 10% or no serum was added along with either DMSO or 10 μM DHPCC-9. The IncuCyte™ Scratch Wound software was used to capture and analyse the pictures. After acquisition, images were combined with ImageJ into a QuickTime movie file.

### Boyden chamber invasion assays

Cell culture invasion inserts of 8 μm pore size (BD Biosciences) were coated with Matrigel (100 μg/cm^2^; BD Biosciences) and incubated for 24 h. The assays were initiated by placing 50 000 cells in DMEM supplemented with 1% BSA together with either 10 μM DHPCC-9 or 0,1% DMSO. Conditioned medium from confluent MG-63 human osteosarcoma cells was used as a chemoattractant to stimulate movement of cells through the *in vitro *basement membranes. Cells were incubated for 72 h, after which insert membranes were fixed for 20 min in 4% paraformaldehyde in PBS and stained with Mayer's haematoxylin (Zymed) for 4 h. Then membranes were washed with PBS and cut from the inserts. Cells on the upper surface of the membrane were wiped off and membranes were mounted with glycerol and PBS (9:1, Merck KGaA). Invaded cells on the lower surfaces of the membranes were counted.

### Statistical analyses

The statistical significance of data was determined by pairwise comparisons between control samples and treated samples by using Student's t-test (Paired Two Sample for Means). Results were interpreted as highly significant*** (p < 0.001), significant** (p < 0.01), weakly significant* (p < 0.05) or not significant^**ns **^(p > 0.05). IC_50 _values of DHPCC-9 in FDCP-1 cells were determined using nonlinear regression fitting with the GraphPad Prism v.5.0. Error bars in all graphs represent SD values.

## Results

### DHPCC-9 abrogates the anti-apoptotic effects of Pim-1 in cytokine-deprived myeloid cells

To identify cellular inhibitors for Pim family kinases, we tested in cell-based assays a panel of small molecule compounds that we and others had recently shown to selectively inhibit Pim kinases under *in vitro *conditions ([21] and unpublished data by the European Union Prokinase Research Consortium). For this purpose, we used IL-3-dependent FDCP1 cell lines stably expressing either neomycin (FD/Neo) or the 44 kD isoform of Pim-1 (FD/Pim44). We had previously shown that survival of FDCP1-derived cell lines in the absence of IL-3 is strictly dependent on continuous expression and activity of either Pim-1 or Pim-2 [18,19,28], so we expected a Pim-specific inhibitor to abrogate the protective effects of Pim kinases. To quantitate the effects of the inhibitors on cellular viability, we used the MTT assay, which measures metabolic activity, but which has been commonly used to analyse e.g. cytotoxic effects of various compounds [30].

**Figure 1:**
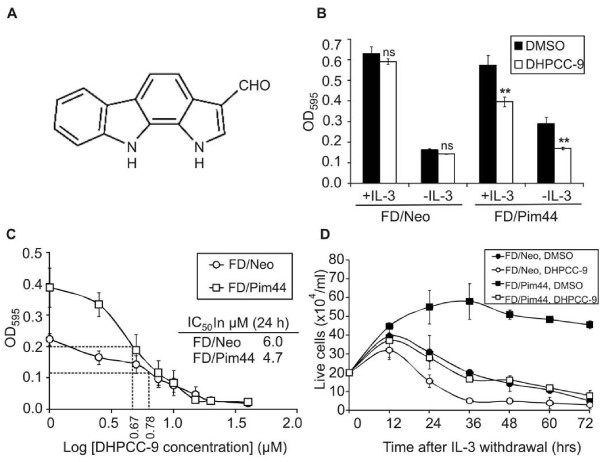
**DHPCC-9 inhibits Pim-1-dependent survival of cytokine-deprived myeloid cells**. **(A) **Schematic structure of 1,10-dihydropyrrolo[2,3-*a*]carbazole-3-carbaldehyde (DHPCC-9). (**B) **FDCP1 cell lines stably expressing neomycin (FD/Neo) or Pim-1 (FD/Pim44) were cultured for 24 h with or without IL-3 in the presence of DMSO or 10 μM DHPCC-9, after which cell viability was analysed by the MTT assay. Graph represents means from three independent experiments with duplicate samples. (**C) **Cells were cultured for 24 h without IL-3 in the presence of increasing concentrations of DHPCC-9. Cell viability was analysed by the MTT assay and IC_50 _values were determined. Points represent means from four independent experiments with duplicate samples. (**D) **Cells grown in the absence of IL-3 were stained with Trypan blue and live cells were counted at the indicated time-points. Points represent means from triplicate determinations from one of two similar experiments.

When FDCP1 derivatives were cultured for 24 h in serum-containing growth medium in the presence of 10 μM compounds dissolved in DMSO, most of them did not have any effects on survival of either FD/Neo or FD/Pim44 cells or reduced viability of both cell lines to a similar extent (data not shown). However, one of the compounds designated DHPCC-9 (1,10-dihydropyrrolo[2,3-*a*]carbazole-3-carbaldehyde; [21]; Figure 1A) completely removed the survival advantage of Pim-1-overexpressing cells (Figure 1B). No major changes were observed in the viability of DHPCC-9-treated FD/Neo cells as compared to DMSO-treated samples, indicating that the inhibitor has no general cytotoxic effects in these cells. By contrast, in FD/Pim44 cells there was a significant decrease in the viability of inhibitor-treated cells in the presence and especially in the absence of IL-3, indicating that DHPCC-9 is indeed a potent cellular inhibitor that can enter the cells and efficiently impair the anti-apoptotic effects of Pim-1.

To determine the effective dosage of DHPCC-9 in FDCP1 derivatives, cells were cultured with increasing concentrations of the inhibitor in the absence of IL-3 and analysed 24 h later by the MTT assay. As shown in Figure 1C, the initially two-fold higher viability rate of FD/Pim44 cells as compared to FD/Neo cells was lost already with fairly low amounts of DHPCC-9, with the viability curves converging at around the 10 μM concentration used in Figure 1B. Moreover, calculation of the IC_50 _values indicated that they were nearly similar in both FD/Neo (6.0 μM) and FD/Pim44 (4.7 μM) cell lines.

To confirm that the results obtained with the MTT assay reflected cell survival, we stained cells with Trypan blue and counted dye-excluding live cells at multiple time-points after withdrawal of IL-3. As shown in Figure 1D, FD/Pim44 cells treated with DMSO were still alive after 72 h, while FD/Neo cells stopped growth and started to die already after 12 h. However, when cells were treated with 10 μM DHPCC-9, the protective effects of constitutively expressed 44 kDa Pim-1 were completely lost and the FD/Pim44 cells behaved like DMSO-treated FD/Neo control cells. In this assay, DHPCC-9 also reduced the viability of FD/Neo cells, which is in line with our previous observations from FDCP1 cells expressing a dominant negative mutant of Pim-1 [28].

### DHPCC-9 inhibits cellular phosphorylation of Pim substrates such as Bad

We had recently shown that DHPCC-9 inhibits kinase activities of all three Pim family proteins under *in vitro *conditions [21]. In order to demonstrate that DHPCC-9 similarly inhibits intracellular activities of Pim kinases, we analysed the phosphorylation status of one of the well-established Pim substrates, the pro-apoptotic Bad protein [18-20]. FD/Neo and FD/Pim44 cells were transiently transfected with a GST-Bad expression vector. Part of the cells were collected 24 h after transfection, while the rest were grown for 8 h in the absence of IL-3, but with increasing concentrations of DHPCC-9.

**Figure 2:**
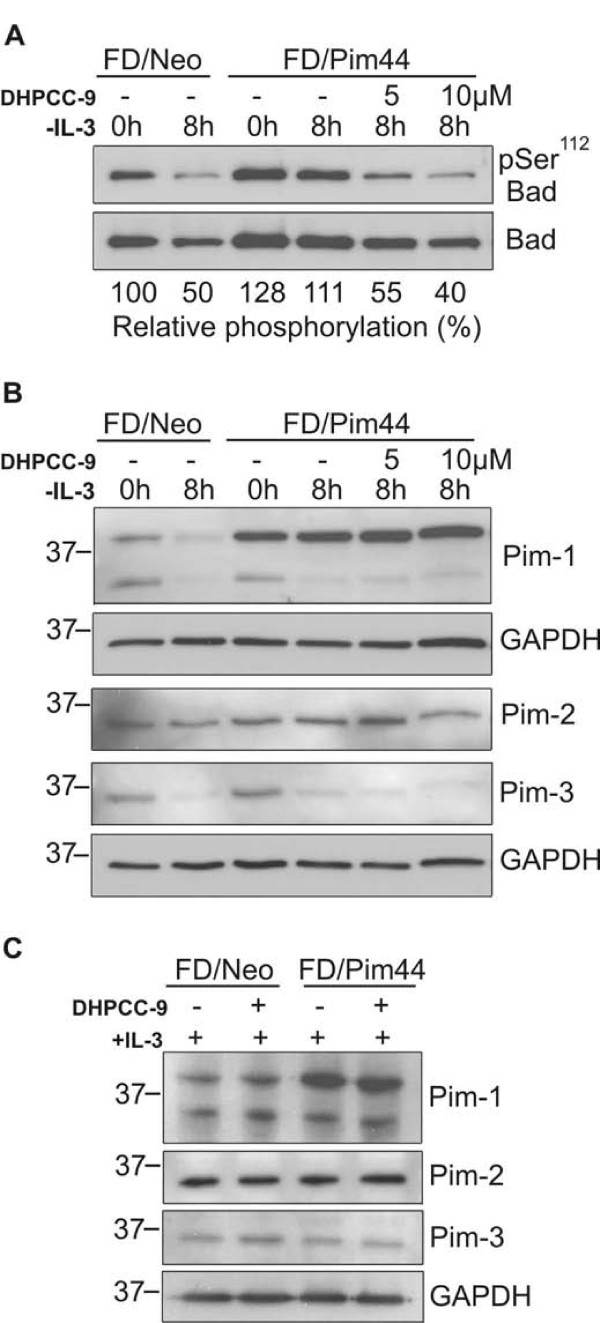
**DHPCC-9 represses intracellular phosphorylation of Bad by Pim kinases**. Cells were transiently transfected with the GST-Bad expression vector. At 24 h after transfection, IL-3 was withdrawn and cells were treated with DMSO or increasing concentrations of DHPCC-9 for indicated time-points. (**A) **GST-Bad was precipitated and its phosphorylation and expression levels were analysed by Western blotting. The intensities of phosphorylated versus total samples of Bad protein were quantitated and the relative intensities of Bad phosphorylation in inhibitor-treated samples as compared to DMSO-treated control sample were calculated. Endogenous Pim expression levels in Bad-transfected **(B) **or untransfected **(C) **FDCP1 derivatives were determined by Western blotting with specific antibodies against distinct Pim family members. GAPDH staining was used as a loading control.

In the absence of IL-3, Ser^112 ^of Bad remained more pronouncedly phosphorylated in FD/Pim44 cells than in FD/Neo cells (Figure 2A), which was well in line with our previous results [19]. However, exposure of FD/Pim44 cells to increasing concentrations of DHPCC-9 led to a significant reduction in the level of Bad Ser^112 ^phosphorylation as compared to Bad expression levels (Figure 2A). We also measured Pim protein levels from the cell lysates and noticed that all three Pim family members were expressed there and that DHPCC-9 did not reduce their expression levels either in the presence or absence of IL-3 (Figure 2B, C). Thus, our results suggest that DHPCC-9 exerts its cellular effects by inhibiting kinase activities of all Pim family members towards their downstream targets. Interestingly, while the endogenous expression levels of both Pim-1 and Pim-3 proteins were significantly reduced by IL-3 withdrawal, the levels of Pim-2 remained unchanged (Figure 2B), suggesting that its expression in FDCP1 cells may be regulated in a distinct fashion from the two other family members.

### Pim kinases promote cancer cell migration and invasion

**Figure 3:**
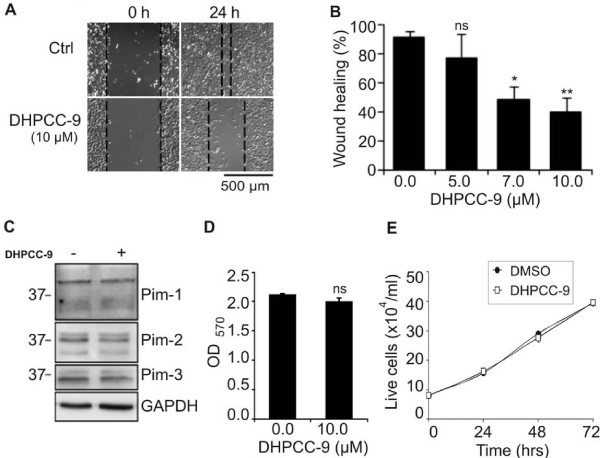
**DHPCC-9 decreases migration of prostate cancer cells without affecting Pim protein levels or cell viability**. PC-3 prostate cancer cells were treated with DMSO or 10 μM DHPCC-9. Two hours later, scratch wounds were made with a 10 μl sterile pipette tip. (**A) **Representative pictures were taken at indicated time-points and analysed. (**B) **Graph represents means from triplicate samples after 24 h incubation. (**C) **Endogenous Pim expression levels were determined by Western blotting. GAPDH staining was used as a loading control. (**D) **Cell viability was analysed by the MTT assay. (**E) **The amount of live cells was determined by Trypan blue staining.

During our recent studies we had obtained hints that Pim kinases may be involved in regulation of cell motility. Migration of cells is important for many physiological processes including embryogenesis, wound healing and immune responses, but it is also essential for tumor angiogenesis and metastasis. Therefore we decided to use DHPCC-9 as a tool to investigate the possibility that Pim kinases affect migration of adherent cancer cells. When we carried out scratch wound assays with PC-3 prostate cancer cells, we noticed that DHPCC-9 decreased the motility of those cells in a dose-dependent fashion (Figure 3A, B), but did not reduce endogenous expression levels of Pim kinases (Figure 3C). Viability of PC-3 cells also remained unaffected by DHPCC-9, as measured both by the MTT assay (Figure 3D) and by Trypan blue staining (Figure 3E).

**Figure 4:**
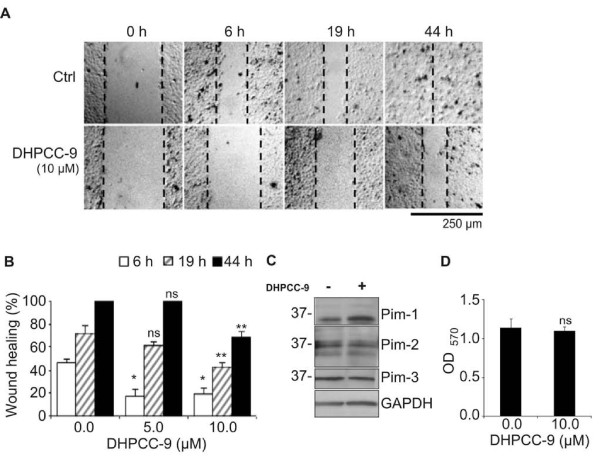
**DHPCC-9 also reduces migration of squamocellular carcinoma cells**. UT-SCC-12-A squamocellular carcinoma cells were treated with DMSO or 10 μM DHPCC-9. Two hours later, scratch wounds were made with a 10 μl sterile pipette tip. (**A) **Representative pictures were taken at indicated time-points and analysed. (**B) **Graph represents means from triplicate samples. **(C) **Endogenous Pim expression levels were determined by Western blotting. GAPDH staining was used as a loading control. (**D) **Cell viability was analysed by the MTT assay.

The inhibitory effects of DHPCC-9 on cell migration were not restricted to PC-3 cells, since similar results were obtained also from UT-SCC-12A squamocellular carcinoma cells (Figure 4A, B). We have previously shown that these cells express high levels of Pim-1 [29], but we now demonstrated that they also express Pim-2 and Pim-3 (Figure 4C). Similarly to PC-3 cells, DHPCC-9 did not decrease either Pim expression levels (Figure 4C) or viability (Figure 4D) of UT-SCC-12A cells.

**Figure 5:**
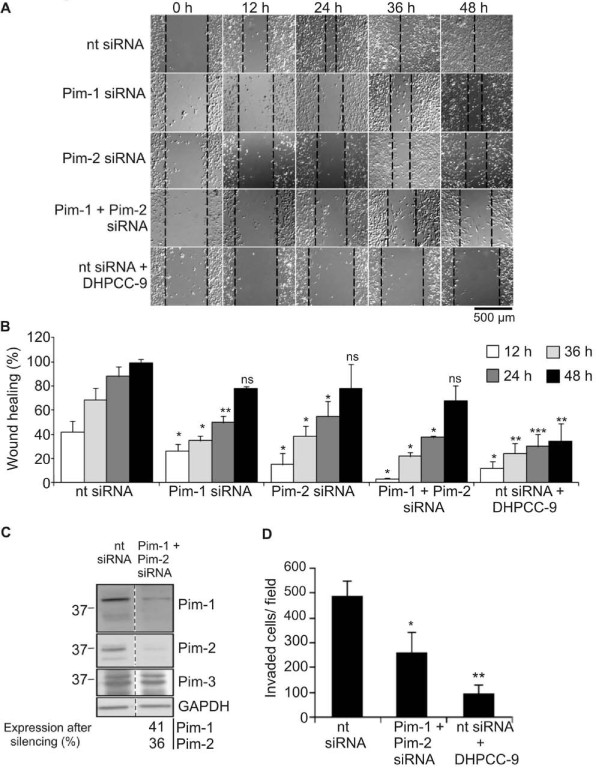
**Silencing and inhibition of Pim kinases both repress migration and invasion of prostate cancer cells**. PC-3 cells were transfected with 100 nM non-targeting (nt) control siRNA or siRNA oligonucleotides targeting *pim *genes, and incubated overnight. (**A) **For wound healing assays, transfected cells were moved onto 24-well plates and allowed to attach for 24 h, after which DMSO or 10 μM DHPCC-9 was added and scratch wounds were made with a sterile 200 μl pipette tip. Representative pictures were taken at indicated time-points and analysed. (**B) **Graph represents means of three independent experiments with duplicate samples. (**C) **Efficiency of Pim kinase silencing was determined by Western blotting. GAPDH staining was used as a loading control. Non-parallel lanes from a single electrophoresis gel are separated by dash lines. (**D) **For invasion assays, transfected cells were placed in invasion inserts together with either DMSO or 10 μM DHPCC-9. MG-63-conditioned medium was used as a chemoattractant to induce invasion. Cells were incubated for 72 h, after which insert membranes were fixed and stained. Invasion assays were repeated for three times and invaded cells were counted from each sample from 10 representative fields. Shown are means of duplicate samples from one representative experiment.

To further prove that the inhibitory effects of DHPCC-9 on cell motility were mediated via specific inhibition of Pim kinase activity, we carried out wound healing assays in PC-3 cells in the presence of short interfering RNAs (siRNAs) targeting either Pim-1, Pim-2 or both. Pim-3 was not targeted, since its expression levels have been reported to be significantly lower in PC-3 cells than those for Pim-1 and Pim-2 [27]. A larger tip was now used to scratch the wounds to facilitate follow-up of the wound healing processes. While wounds in control cells transfected with non-targeting (nt) siRNA healed within 48 h, those expressing the Pim-specific siRNAs recovered significantly more slowly (Figure 5A, B). These results were not due to differences in cell viability, as confirmed by the MTT assay (data not shown). Most striking reduction in migration was observed when both Pim-1 and Pim-2 were silenced. In fact, the effects of simultaneously silenced Pim-1 and Pim-2 were initially comparable to those observed in control transfectants treated with DHPCC-9. However, the effects of DHPCC-9 were more sustainable, since they were visible even after the 48 h time-point (Figure 5A, B and data not shown). Here it should also be noted that the siRNA transfection efficiency was approximately 25% (data not shown), so only partial silencing of Pim kinases was received, as demonstrated by Western blotting (Figure 5C).

To follow-up the wound healing process in more detail and to compare the effects of DHPCC-9 in the presence or absence of serum, we used the IncuCyte™ imaging system with a scratch wound application. In the presence of 10% serum, we obtained very similar results as in manual experiments, while removal of serum reduced cell migration rates (Additional File 1). Due to the lower migration rates of serum-deprived cells, it was possible to follow the movements of individual cells in movies constructed from the slides produced by the IncuCyte™ (Additional File 2). There DHPCC-9-treated cells seemed to have totally lost their ability to move, whereas only a few of the control cells seemed equally immotile.

To determine whether Pim kinases are able to affect invasive properties of PC-3 cells, we carried out Boyden chamber assays with PC-3 cells that had been transfected with non-targeting or Pim-specific siRNAs and treated with either DMSO or DHPCC-9. Three days later, cells were fixed and stained to facilitate counting of invaded cells. As summarized in Figure 5D, silencing of Pim-1 and Pim-*2 *reduced the rate of invasion of PC-3 cells. However, even more striking effects were obtained in the presence of DHPCC-9, which allowed only a minority of the cells to move through the membranes.

**Figure 6:**
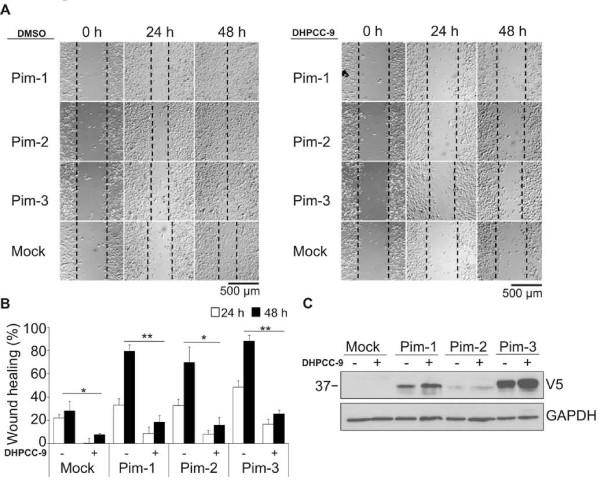
**Pim kinase overexpression promotes migration of prostate cancer cells**. PC-3 cells were transiently transfected with an empty vector (Mock) or vectors expressing V5-tagged Pim-1, Pim-2 or Pim-3. DMSO or 10 μM DHPCC-9 were added 24 h after transfection. The wound healing assays were initiated 2 h later by scratching the wounds with a sterile 200 μl pipette tip. (**A) **Shown are representative pictures from three time-points. (**B) **Graph represents means of triplicate samples. (**C) **Levels of overexpressed Pim kinases were determined by Western blotting with anti-V5 antibody. GAPDH staining was used as a loading control.

To obtain even more evidence to support our conclusion that Pim kinases enhance cell motility, we transiently overexpressed them in PC-3 cells and subjected cells to wound healing assays in the absence or presence of DHPCC-9. Indeed, cells overexpressing any of the three Pim family members migrated remarkably faster than mock-transfected control cells (Figure 6A left panel). Furthermore, DHPCC-9 reduced the migration rates of Pim-transfected cells almost to the levels of the control cells (Figure 6A right panel). As summarized in Figure 6B, the enhancing effects of Pim kinases on cell migration were comparable with each other, even though there was variation in their overexpression levels (Figure 6C). As in the case of endogenous Pim expression, DHPCC-9 did not reduce expression of the V5-tagged Pim proteins (Figure 6C). Moreover, when transfected cells were subjected to MTT assay, no major changes were observed (data not shown), indicating that the effects of Pim kinases on PC-3 cell motility were not due to enhanced proliferation. In conclusion, our data with the Pim inhibitor as well as the Pim-specific siRNAs and overexpression constructs suggest that the expression and activity of Pim family kinases are essential for both migration and invasion of adherent cancer cells.

### DHPCC-9 inhibits pro-migratory effects of one of the Pim substrates, NFATc1

We next wanted to address the possibility that the effects of Pim kinases on cell motility are at least partially mediated via NFATc transcription factors that we have previously identified as Pim targets [16]. This was of interest, since there is endogenous NFATc activity in PC-3 cells [31] and since NFATc factors have recently been implicated in cancer cell migration and invasion [32].

**Figure 7:**
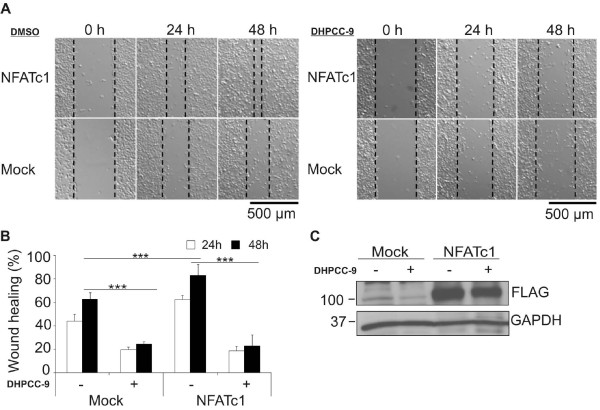
**Enhancing effects of NFATc1 on migration of prostate cancer cells are abolished by DHPCC-9**. PC-3 cells were transiently transfected with an empty vector (Mock) or a vector expressing FLAG-tagged NFATc1. DMSO or 10 μM DHPCC-9 were added 24 h after transfection. The wound healing assays were initiated 2 h later by scratching the wounds with a 10 μl sterile pipette. (**A) **Shown are representative pictures from three time-points. (**B) **Graph represents means of two similar experiments with triplicate samples. (**C) **Overexpression of NFATc1 was confirmed by Western blotting with anti-FLAG antibody. GAPDH staining was used as a loading control.

When we transiently overexpressed FLAG-tagged NFATc1 in PC-3 cells and subjected cells to wound healing assays in the absence or presence of DHPCC-9, we observed that cells overexpressing NFATc1 healed significantly faster than mock-transfected cells (Figure 7A-B). Yet cell viability or Pim expression levels were not affected by NFATc1 overexpression, as demonstrated by Trypan blue staining and Western blotting, respectively (data not shown), indicating that the effects of NFATc1 on PC-3 cell migration were not due to enhanced proliferation or Pim expression. More interestingly, the Pim kinase inhibitor DHPCC-9 was able to reduce migration of NFATc1-transfected cells to the same extent as of the control cells (Figure 7A-B), while it did not significantly affect the expression levels of FLAG-tagged NFATc1 (Figure 7C). Thus, these results suggest that Pim kinases promote cancer cell migration by regulating activity of NFATc transcription factors.

## Discussion

In this study, we have characterized the biological effects of 1,10-dihydropyrrolo[2,3-*a*]carbazole-3-carbaldehyde (DHPCC-9) and demonstrated that it is an efficient cell-permeable inhibitor targeting all human or murine Pim family kinases. We have shown that DHPCC-9 abrogates the anti-apoptotic effects of Pim-1 in cytokine-deprived FDCP1 myeloid cells, while it does not display general cytotoxicity at the micromolar concentrations used. DHPCC-9 treatment also inhibits intracellular phosphorylation of Pim substrates such as Bad. Furthermore, we have been able to use DHPCC-9 as a tool to reveal a novel function for Pim kinases in regulation of motility of adherent cancer cells. Treatment with DHPCC-9 significantly slows down migration of both PC-3 prostate cancer cells and UT-SCC-12A squamocellular carcinoma cells, but does not significantly affect metabolic activity or viability of these cells or their levels of Pim expression. In addition, DHPCC-9 efficiently inhibits invasion of PC-3 cells through matrigel.

The observed effects of DHPCC-9 are most likely dependent on its ability to inhibit Pim kinase activity, since similar results were obtained by silencing Pim expression by RNA interference. In addition, overexpression of any Pim family member has opposite effects by enhancing cancer cell motility, providing further proof that Pim kinases are potent regulators of cellular processes involved in migration and/or invasion.

In all of our assays, the effects of DHPCC-9 were more pronounced than those of Pim-specific siRNAs, most likely due to the longer half-life and better cellular penetrance of the inhibitor. Indeed, when the stability of DHPCC-9 dissolved in the growth medium of PC-3 cells was analysed by liquid chromatography, no detectable degradation of the compound was observed within the time-periods used in our assays (unpublished data). By contrast, siRNAs affect only a portion of cells depending on transfection efficiency and their transient effects are rapidly diluted within the few days after transfection. Here it should also be noted that while DHPCC-9 is able to target all the three Pim kinases endogenously expressed in PC-3 cells, Pim-3 remains active in cells treated with the combination of siRNAs against Pim-1 and Pim-2. Yet in PC-3 cells the expression levels for Pim-3 have been reported to be significantly lower than for the two other family members [27]. Thus, even though all Pim kinases may be equally capable of enhancing cell motility, the magnitude of their redundant effects is likely to depend on their dosage in each cell type.

An intriguing feature of Pim family kinases is that they are constitutively active with a unique hinge region containing a proline residue, which allows only one hydrogen bond to be formed with ATP [3]. According to crystallization studies [21], DHPCC-9 does not bind to the hinge region as some other ATP-mimetic Pim inhibitors do [33,34]. By contrast, it competes with ATP for binding to the conserved lysine residue corresponding to Lys67 in Pim-1 [21]. This residue is known to be critical for Pim kinase activity, since its mutation e.g. to methionine results in production of a kinase-deficient protein [16].

Under *in vitro *conditions, we have previously demonstrated that DHPCC-9 is highly potent against Pim-1, Pim-2 and Pim-3 kinases with very low IC_50 _values of 12, 51 and 10 nM, respectively [21]. It is also highly selective, since with the 10 μM concentration used in most of our cellular assays, the *in vitro *activities of Pim-1, Pim-2 and Pim-3 kinases are inhibited by 98, 93 and 99%, respectively, while all the other 88 kinases tested remain fairly active ([21] and unpublished data). Yet we cannot fully exclude the possibility that some of the observed effects of DHPCC-9 are enhanced by its moderate ability to inhibit also other kinases. Interestingly, DHPCC-9 seems to target Pim-1 and Pim-3 slightly more efficiently than Pim-2. This selectivity is in line with similar observations from several other Pim inhibitors, as recently discussed [13]. Even though Pim-1 and Pim-3 are more closely related to each other than to Pim-2, structural models are unable to explain the observed differences in their sensitivities.

In cell-based assays, the activities of inhibitors are not expected to be as high as under *in vitro *conditions with purified compounds. Thus, the micromolar IC_50 _values observed in FD/Neo (6.0 μM) and FD/Pim44 (4.7 μM) cells can be considered promising, especially since they were obtained using complete medium containing 10% serum. Cytotoxicity assays with some other Pim inhibitors have been carried out in the presence of lower or no serum levels [22,27], which probably has decreased the viability of the cells and also enhanced bioavailability of the inhibitors and consequently lowered their IC_50 _values. Indeed, more pronounced effects were observed also in our assays with DHPCC-9, when cells were grown in the absence of serum.

Yet it is likely that the efficiency of the Pim inhibitor can be further improved by using DHPCC-9 as a scaffold for production of additional, more potent derivatives, which could be useful not only as research tools, but also as lead compounds in development of drugs against Pim-overexpressing tumors. Since DHPCC-9 has been shown to be highly efficient in reducing the motility of Pim-overexpressing cancer cells, derivatives of DHPCC-9 might be able to prevent tumor metastasis and/or angiogenesis. Moreover, since we and others have recently shown that Pim kinases are involved in development of resistance against radiation therapy or chemotherapy [29,35], combinations of Pim inhibitors together with other anticancer therapy methods are expected to lead to most efficient therapeutic approaches.

Even though Pim kinases have been implicated to have prognostic roles in several types of solid cancer, there is still controversy in the literature on whether or not high levels of Pim expression are of disadvantage for prostate cancer patients (reviewed in [12,13]). This may be partly due to heterogeneity of the samples and to the fact that in none of the studies published so far have expression levels for all three Pim kinases been analysed in parallel. It is also clear that overexpressed Pim kinases alone are unable to transform cells, but require collaboration e.g. with Myc oncoproteins. Interestingly in this regard, coexpression of Pim-1 and c-Myc in human prostate tumors has recently been associated with higher Gleason grades than overexpression of either one alone, suggesting that these oncoproteins synergize to induce advanced prostate carcinoma [36].

While our work was in progress, silencing of Pim-3 was reported to reduce endothelial cell spreading, migration and vascular tube formation [37], providing further support to our hypothesis that Pim kinases can stimulate metastatic and/or angiogenic potential of cancerous cells. In addition, Pim-1 but not Pim-2 was shown to regulate homing and migration of bone marrow cells, possibly via phosphorylation-mediated modification of CXCR4 expression on cell surface [38]. Yet the exact substrates and signalling pathways that all three Pim kinases regulate to enhance motility of adherent cancer cells remain to be elucidated. Interestingly, the NFATc transcription factors that we have previously identified as Pim targets [16] have recently been implicated in tumor cell migration and invasion as well as tumor angiogenesis (reviewed in [39]). Constitutively active NFATc isoforms have been shown to promote induction and progression of both hematological malignancies and solid tumors by driving synthesis and secretion of pro-angiogenic factors (e.g. cyclo-oxygenase 2 and tissue factor) as well as factors promoting cell motility (e.g. lysophosphatic acid and prostaglandin E2). Thus, these NFATc-dependent effects are expected to be enhanced in tumor cells overexpressing Pim kinases. Indeed, our data indicate that Pim inhibitors can block the pro-migratory effects of NFATc factors in prostate cancer cells, suggesting that regulation of NFATc activity may be one of the mechanisms how Pim kinases promote cancer cell motility.

## Conclusions

Altogether, our data indicate that Pim kinases can stimulate migration and invasion of adherent cancer cells, possibly via NFATc factors. Therefore, the novel Pim kinase inhibitor DHPCC-9 is not only an efficient tool for Pim research, but also a promising compound for cancer drug development and could be targeted especially to inhibit invasiveness of Pim-overexpressing cancer cells.

## Competing interests

The authors declare that they have no competing interests.

## Authors' contributions

NMS designed and carried out the migration and invasion analyses and drafted the manuscript. RLV analysed inhibitory activities of various compounds in cell-based assays and helped to draft the manuscript. EMR helped in phosphorylation and wound healing assays, JAS in image analyses and SSV in invasion assays. FA, PM and MP synthesized the DHPCC-9 inhibitor, tested its stability and provided sufficient amounts of it for the biological experiments. PJK conceived and coordinated the study, designed the cell-based assays to recognize cellular inhibitors for Pim kinases and edited the manuscript. All authors read and approved the final manuscript.

## Supplementary Material

Additional file 1 **DHPCC-9 decreases PC-3 cell migration both in the presence and absence of serum**. PC-3 cells were plated on 24-well Essen ImageLock plates. Scratch wounds were made 24 h later by the Essen WoundMaker™ with a sterile 10 μl Eppendorf pipette tip. Thereafter fresh culture medium containing 10% or no serum along with either DMSO or 10 μM DHPCC-9 was added. Essen IncuCyte™ Scratch Wound software was used to capture pictures and to analyse the wound confluences. (**A) **Graph represents means of triplicate samples. (**B) **Shown are representative pictures from five time points. Click here for file

Additional file 2 **DHPCC-9 decreases PC-3 cell migration both in the presence and absence of serum**. A movie file was produced from PC-3 scratch wound samples described in the Additional File 1. Pictures taken by the IncuCyte™ Scratch Wound software were combined into a QuickTime movie file with ImageJ. Shown are representative wound healing movies of PC-3 cells treated with DMSO **(A, C) **or DHPCC-9 **(B, D) **and cultured in medium containing 10% serum **(A, B) **or no serum **(C, D)**. Click here for file
