# Supplementary figures and images for: Pim-selective inhibitor DHPCC-9 reveals Pim kinases as potent stimulators of cancer cell migration and invasion
Source: Mol Cancer. 2010 Oct 19;9:279. doi: 10.1186/1476-4598-9-279 (PMC2978147; doi:10.1186/1476-4598-9-279)

# Additional File 1

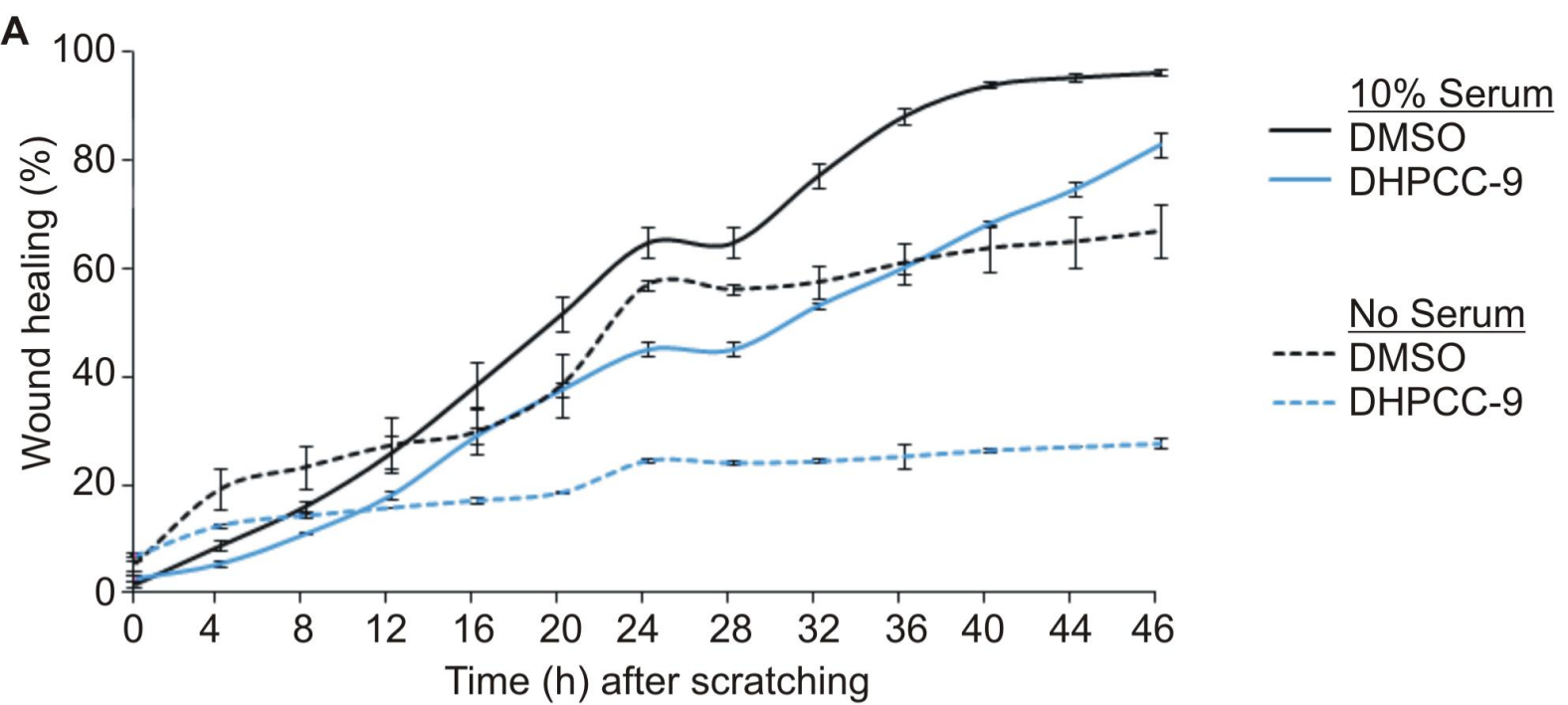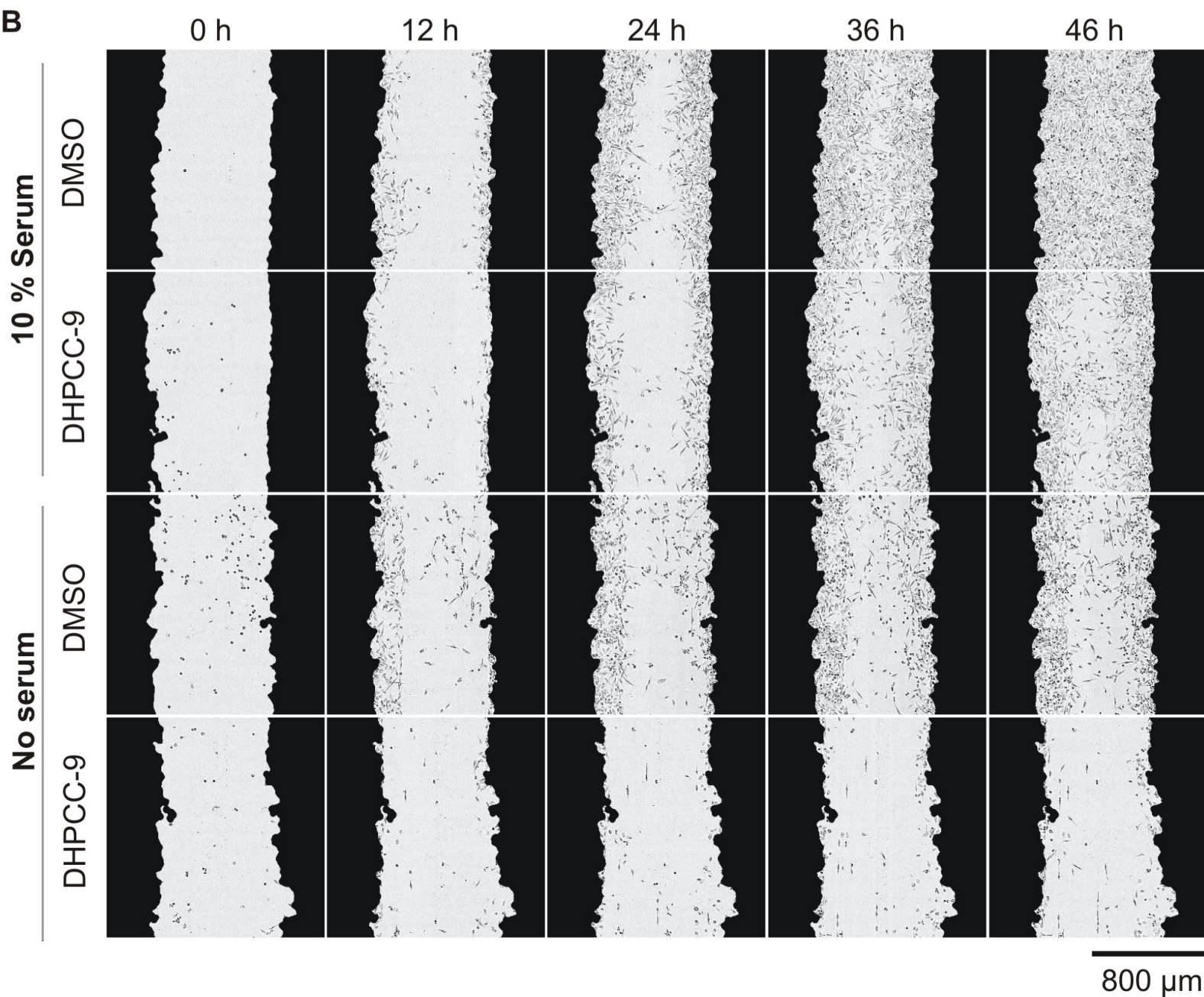

Supplement: Additional file 1 — DHPCC-9 decreases PC-3 cell migration both in the presence and absence of serum. PC-3 cells were plated on 24-well Essen ImageLock plates. Scratch wounds were made 24 h later by the Essen WoundMaker™ with a sterile 10 μl Eppendorf pipette tip. Thereafter fresh culture medium containing 10% or no serum along with either DMSO or 10 μM DHPCC-9 was added. Essen IncuCyte™ Scratch Wound software was used to capture pictures and to analyse the wound confluences. (A) Graph represents means of triplicate samples. (B) Shown are representative pictures from five time points. [file 1476-4598-9-279-S1.PDF]
